# Spatially Resolved Dynamics of Cobalt Color Centers in ZnO Nanowires

**DOI:** 10.1002/advs.202205304

**Published:** 2022-11-20

**Authors:** Christian T. Plass, Valentina Bonino, Maurizio Ritzer, Lukas R. Jäger, Vicente Rey‐Bakaikoa, Martin Hafermann, Jaime Segura‐Ruiz, Gema Martínez‐Criado, Carsten Ronning

**Affiliations:** ^1^ Institut für Festkörperphysik Friedrich‐Schiller‐Universität Jena Max‐Wien‐Platz 1 07743 Jena Germany; ^2^ ESRF – The European Synchrotron 71 Avenue des Martyrs Grenoble 38043 France; ^3^ Instituto de Ciencia de Materiales de Madrid (CSIC) Consejo Superior de Investigaciones Científicas Calle Sor Juana Inés de la Cruz 3, Cantoblanco Madrid 28049 Spain

**Keywords:** color centers, nanowires, nano‐X‐ray excited optical luminescence (XEOL), nano‐XRF, time‐resolved X‐ray excited optical luminescence (XEOL), ZnO

## Abstract

The dynamics of color centers, being a promising quantum technology, is strongly dependent on the local environment. A synergistic approach of X‐ray fluorescence analysis and X‐ray excited optical luminescence (XEOL) using a hard X‐ray nanoprobe is applied. The simultaneous acquisition provides insights into compositional and functional variations at the nanoscale demonstrating the extraordinary capabilities of these combined techniques. The findings on cobalt doped zinc oxide nanowires show an anticorrelation between the band edge emission of the zinc oxide host and the intra‐3d cobalt luminescence, indicating two competing recombination paths. Moreover, time‐resolved XEOL measurements reveal two exponential decays of the cobalt luminescence. The fast and newly observed one can be attributed to a recombination cascade within the cobalt atom, resulting from direct excitation. Thus, this opens a new fast timescale for potential devices based on cobalt color centers in ZnO nanowires in photonic circuits.

## Graphical abstract

Zinc oxide nanowires are implanted with cobalt atoms to create color centers. These are investigated using temporally and spatially resolved X‐ray excited optical luminescence combined with X‐ray fluorescence. The results reveal an anticorrelation of band edge emission and cobalt luminescence, indicating competing recombination paths. Two exponential decays are found, with a new fast decay attributed to the direct excitation.

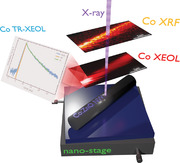

## 1 Introduction

Color centers in semiconductors have gained enormous importance for quantum technologies lately. They provide peculiar properties with enormous potential in this field.^[^ ^1^, ^2^, ^3^, ^4^, ^5^, ^6^ ^]^ Their main characteristics are the capability to act as a qubit and to offer high quality single photon sources. Such quantum emitters have been observed in several material systems,^[^ ^7^, ^8^ ^]^ as the generation of single photons was demonstrated in quantum dots,^[^ ^9^ ^]^ single molecules^[^ ^10^ ^]^ or, most prominent, by defect‐related centers in wide‐bandgap semiconductors.^[^ ^8^, ^11^, ^12^, ^13^ ^]^ A promising material with suitable properties for defect‐related emission in a broad visible range is zinc oxide (ZnO).^[^ ^14^ ^]^ Color centers in ZnO can be achieved by doping it with rare earth elements or transition metals, such as copper, nickel, iron, or cobalt.^[^ ^15^, ^16^, ^17^, ^18^ ^]^ In particular, the transition metals provide fast decay times and, therefore, fast optical responses. Embedding such color centers into nanowires additionally offers the potential of a coupling platform between single photon emitters and photonic circuits, because nanowires provide waveguiding as well as a cavity for the emitted photons. Hence, investigating the photocarrier dynamics, with respect to the local environment, is of great importance.

**Figure 1:**
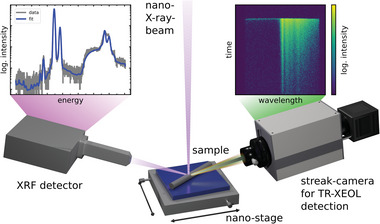
Schematic layout of the experimental setup to simultaneously measure nano‐XRF and nano‐TR‐XEOL. A representative XRF spectrum for a single spot is shown on the left side along with a fit, while a spectrally and temporally resolved streak image of the XEOL signal is shown on the right side.

In this study, high spatial resolution synchrotron‐based methods were used to evaluate how the carrier dynamics and the luminescence is influenced by the elemental composition and the local environment of cobalt centers in ZnO nanowires. Previously, such systems were investigated with different techniques, such as photoluminescence or cathodoluminescence measurements.^[^ ^15^, ^16^, ^17^, ^19^ ^]^ However, these techniques lack crucial complementary information, in terms of spatial or compositional variations of the system, respectively. Simultaneous and spatially resolved measurements of the X‐ray fluorescence (nano‐XRF) and the X‐ray excited optical luminescence (nano‐XEOL) provide both information at once, as also demonstrated by Lin et al.^[^ ^20^, ^21^, ^22^, ^23^ ^]^ Additionally, a streak camera system was used to investigate the luminescence transients and dynamics of the color centers (TR‐XEOL), as depicted in **Figure** **1**. This approach provides unique insights into the different carrier decay paths of the intrashell luminescence of the cobalt atoms incorporated into the lattice of ZnO nanowires.

## 2 Results and Discussion

### 2.1 Elemental Distribution

**Figure 2:**
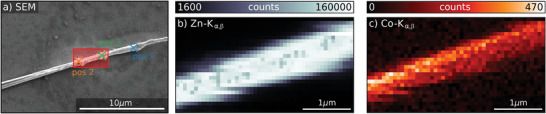
a) Scanning electron microscope image of the investigated ZnO nanowire doped with a nominal Co concentration of 0.7 at% taken after X‐ray irradiation. The red rectangle marks the region investigated by XRF and XEOL. The crosses mark the positions where the time‐resolved XEOL measurements were acquired. Elemental distribution maps obtained from nano‐XRF data for b) Zn and c) Co.

A scanning electron microscope (SEM) image of one representative ZnO nanowire with a nominal Co doping concentration of 0.7 (at% is shown in **Figure** **2**a) and was taken after the X‐ray nano‐analysis. The sample areas for the combined XRF and XEOL studies are indicated in Figure 2. The red rectangle marks the area for spatially resolved measurements, while the crosses indicate the positions for high‐resolution temporally resolved XEOL measurements.

The maps of the distribution of Zn and Co were extracted from the obtained XRF data and are shown in Figure 2b,c, respectively. A comparison of both maps (see overlaid maps in Figure S4, Supporting Information) shows that the concentration of the Co atoms is higher at the top side of the nanowire. This is in good agreement with the implantation profile (see Figure S1, Supporting Information), as the peak concentration is at a depth of about 180 nm compared to the thickness of 650–800 nm of the nanowire. The Co concentration obtained by fitting the XRF spectra at the point with the highest Co XRF signal intensity is about 0.5 at%, and therefore; in good agreement with the SRIM calculations of 0.7 at%.

Previous nano‐XRF investigations have shown a homogenous distribution of Zn and Co over the whole nanowire.^[^ ^24^ ^]^ This finding is in good agreement with the present study as well, because the nanowires in reference 24 were 300 nm in diameter and, thus, thinner. Generally, the substitutional incorporation of Co ions implanted into the ZnO host matrix of nanowires has already been shown by nano‐EXAFS.^[^ ^25^ ^]^ Cobalt atoms incorporate at Zn lattice sites after ion implantation, inducing structural disorder that can be fully recovered through a thermal annealing process.^[^ ^25^, ^26^ ^]^ Therefore, these nanowires can be assumed to have high structural order without significant lattice distortion of interatomic distances after annealing.^[^ ^25^, ^26^, ^27^, ^28^ ^]^

### 2.2 Spatially Resolved XEOL

**Figure 3:**
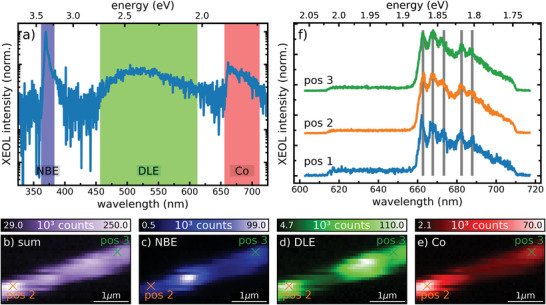
XEOL measurements of the nanowire area shown in Figure 2 (red square). a) Sum spectrum of the XEOL acquired over the entire measurement area with the different characteristic bands indicated by colored rectangles. Nano‐XEOL intensity maps of the measured area for b) the full spectral width, c) the NBE, and d) defect emission of the ZnO host, as well as e) the luminescence of the Co atoms. f) XEOL spectrum in the spectral range of the Co emission detected with the streak camera at three positions marked in Figure 2a and in (b)–(e). The gray vertical lines indicate the positions of different peaks resulting from phonon replica, while the first one represents the zero phonon line.

Corresponding nano‐XEOL measurements are presented in **Figure** **3**, which were taken simultaneously with the XRF data shown in Figure 1b,c. The XEOL spectrum integrated over the whole area is displayed in Figure 3a. It shows the three characteristic bands of Co implanted ZnO nanowires: the near band emission (NBE, blue), the deep level emission (DLE, green), and the intra‐3d shell Co luminescence (red).^[^ ^15^, ^16^ ^]^ Moreover, the spectrally integrated and spatially resolved XEOL yield is depicted in Figure 3b. By comparing the XEOL maps with the Zn map in Figure 2 (see overlaid maps in Figure S5, Supporting Information), it can be concluded that the complete nanowire shows optical luminescence when it is hit by the X‐ray nanobeam; however, the XEOL spectra vary as a function of the position. The intensity of the different spectral components: NBE, DLE, and Co luminescence, over the scanned sample area is shown in the maps in Figure 2c–e, respectively. Our results evidently reveal that the luminescence of NBE is anticorrelating with DLE and Co emission, likely due to the origin of the two emission paths. The NBE results from the recombination of excitons, while the DLE and Co emission originate from intrinsic and extrinsic defect states within the bandgap, respectively, providing additional carrier recombination paths. Thus, these optical emissions are competing channels and anticorrelate spatially due to structural variations in the nanowire. The defects causing the spectral deviations were likely introduced by slight inhomogeneous implantation, due to a tilt in respect to the ion beam, the varying diameter or shielding by other nanowires upon the implantation process. We further observe that the main features in the DLE intensity map do not correlate with the locations of the performed point measurements, where the material is exposed to a high X‐ray dose, suggesting negligible effects by the intense X‐ray nanobeam.

Comparing the spatial optical emission characteristics with the Co distribution (see overlaid maps in Figure S6, Supporting Information) gives insights on the influence of the incorporated Co atoms. As expected, the Co luminescence is correlating with the Co distribution, and we find a clear anticorrelation trend with both the NBE and DLE signals. This trend indicates a higher amount of nonradiative recombination centers in the vicinity of cobalt atoms. Apparently, the overall crystal quality can be restored by thermal annealing; however, a local higher defect density might remain in the implantation volume.

Three spectra of the Co luminescence were taken with high resolution at 7 K during the point measurements and are shown in Figure 3f. The coordinates at which these measurements were conducted can be found in Figure 2a. The spectra clearly show the zero phonon line (ZPL) of the Co color center being at 1.8708 eV together with the phonon sideband and its well‐resolved phonon replica lines. The phonon modes, with their energy positions being independent of both the Co concentration and the nanowire diameter, are assigned in **Table** **1** according to refs. [30] and [31]. Remarkably, the E_2_^low^, LA, E_2_^high^, and LO modes can be clearly distinguished and assigned to 12.5, 30.1, 54.2, and 66.7 meV, respectively. Furthermore, spectra of different nanowires and Co concentrations can be found in Figure S7 and S8 (Supporting Information). The intensity of the different lines varies with the Co concentration, as expected. The ZPL is higher relative to the other lines for lower Co concentrations, while for higher concentrations the phonon‐replica increase relatively. In the former case the crystal quality is kept higher, because fewer defects are introduced upon implantation.

**Table 1:** Positions of the different phonon lines found in Figure 3f (with an uncertainty of ≈1 meV, resulting from an average of all measured nanowires) and the differences between the respective lines. The phonon modes are assigned according to refs. [29] and [30]

	ZPL	1st line	2nd line	3rd line	4th line
Position [eV]	1.871	1.857	1.841	1.817	1.803
Difference to ZPL [meV]	0	14	30	54	68
Possible phonon modes	ZPL	E_2_^low^ 12.5 meV	LA 30.1 meV	E_2_^high^ 54.2 meV	LO_2_ 66.7 meV

### 2.3 Temporally Resolved XEOL

**Figure 4:**
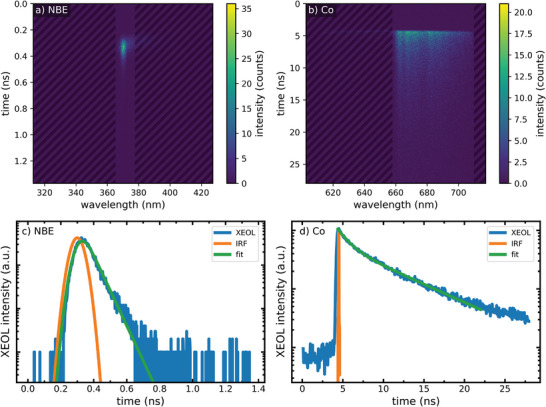
Time‐resolved nano‐XEOL measurements of single Co doped ZnO nanowires at position 2 indicated in Figure 2a detected with the streak camera. Spectrally resolved streak data of a) the ZnO NBE emission and b) the Co luminescence. XEOL signal integrated along the wavelength axis within the marked range for c) the ZnO NBE emission and d) the Co luminescence. The data, the instrument response function, and the resulting fit are shown.

**Figure** **4** shows the time‐resolved nano‐XEOL spectra of both the NBE and Co luminescence, obtained with a streak camera. The luminescence is shown as a function of time and spectral wavelength in Figure 3a,b; while respective scans along the time axis of the detected signals are shown in Figure 3c,d. The decay time of the NBE can be determined to be slightly larger than the instrument response function (IRF) in the 16‐bunch mode with a gaussian profile of a FWHM of 85 ps. The fit (green line) to the NBE transient as well as the logarithmic scale of the plot show a monoexponential decay. The decreased steepness for later times is caused by the background. The resulting decay times differ for certain nanowires. Two wires (one from each preparation set) have a decay time of ≈58 ps, while the other wires show a decay time of around 11 ps (see Figure S9, , Supporting Information). The main difference between these wires can be found in the intensity of the NBE signal. It is significantly higher for the longer decay times, which can be attributed to different aspects. Most likely, the fitting routine cannot be applied for too low intensities and is therefore not reliable in this regime. On the other hand, it might be possible that a higher defect concentration results in a reduced intensity and a quenching of the decay times as well. These decay times are in good agreement with values reported previously by different groups using time‐resolved PL.^[^ ^17^, ^31^ ^]^

The DLE signal could not be monitored in our measurements, indicating a decay time that is much longer than the period of the synchrotron bunches. This is supported by the signal detected between two pulses (see Figure S10, Supporting Information). However, a faster decay has been found by other groups,^[^ ^32^ ^]^ potentially also suggesting a too low signal intensity.

Remarkably, the temporal decay of the Co luminescence together with its phonon sidebands could also be clearly detected by our setup, as shown in Figure 3b,d, even though its intensity is orders of magnitude smaller than the NBE luminescence. The low intensity stems from the 3d intrashell transition of the implanted Co atoms; hence, each atom can emit only one photon per excitation. Here, the number of excited atoms is limited by the probed volume of the nanofocused X‐ray beam. The number of Co atoms contributing to the XEOL signal can be estimated by the concentration of Co atoms, the diameter of the ZnO nanowire, and the X‐ray beam size, while additionally considering that the excited volume might be larger. A rough estimation of the order of magnitude of contributing Co color centers results in 10^5^–10^6^. A minimum number of color centers can be estimated at 10^3^, highlighting the excellent sensitivity of our time‐resolved XEOL setup.

The decay time of the intrashell Co luminescence was analyzed as depicted in Figure 4d. The excitation with the X‐ray pulse is significantly shorter than the decay of the luminescence, which consists out of two exponential decays: a fast decay of about 1.7 ns and a slower decay of about 7 ns. The latter is in very good agreement with literature,^[^ ^17^ ^]^ while the fast one has not been observed, yet. We propose that the fast decay is associated with a mechanism that can only be accessed via excitation with X‐rays. The fast decay might originate from the direct excitation of Co atoms: the inner‐shell ionization of Co atoms upon the absorption of an X‐ray photon is followed by X‐ray fluorescence and Auger decay steps. Subsequently, the electron cascade events will excite 3d electrons of the Co atoms, which will eventually yield the optical luminescence. On the other hand, the slow decay of 7 ns originates from electrons, which have been excited into the conduction band of the ZnO semiconductor. Subsequent energy transfer to the Co atoms results in their excitation and in the following intrashell luminescence emission. These processes have been well investigated in typical PL experiments, where the same process occurs with the valence band electrons.^[^ ^16^, ^17^ ^]^ Furthermore, we have determined the transients of only the zero‐phonon line and separately each phonon replica (see Figure S11, Supporting Information). All lines also show two similar decay times indicating that the interaction of the emitting Co atom with phonons has no influence on the recombination probability.

## 3 Conclusion

In conclusion, the optical luminescence and the composition of single cobalt doped nanowires were investigated with a combinatory approach using simultaneous XRF, XEOL, and TR‐XEOL with a nanoscale lateral resolution using a hard X‐ray nanoprobe. The distribution of cobalt atoms along the nanowire was shown to correspond to the expected one given by the ion implantation parameters. An anticorrelation of the NBE and the intra‐3d luminescence of the cobalt atoms was revealed, while the cobalt distribution correlates with the corresponding optical emission. This indicates that these two recombination channels are counteracting each other, likely because excitons can transfer their energy and recombine at the cobalt dopants. In addition, the phonon interaction of the emitting cobalt atoms could be observed, and the corresponding phonon lines were properly assigned. Moreover, the dynamics of the different spectral features of the nanowire emission were investigated. A decay time of 62 ps for the NBE was obtained, which is in the range of the exciting X‐ray pulse. In contrast, the intra 3d shell cobalt luminescence shows two different carrier recombination paths with decay times of 1.7 and 7 ns, independent of the local cobalt concentration. The slower decay component is attributed to the energy transfer of excited electrons in the conduction band of ZnO to the cobalt color centers. On the other hand, the faster decay is attributed to the direct excitation of core electrons of the cobalt atoms by the X‐rays. Thus, we can propose a way to increase the recombination dynamics of cobalt color centers by a factor of four by direct excitation with an intense X‐ray beam. This suggests that an overall faster decay should be achieved also for resonant excitation of the Co levels. Therefore, the possible timescales of potential devices based on cobalt color centers in ZnO nanowires are faster than expected, regardless of the cobalt content excitation position and the location at which the nanowire is excited, because they could be operated under resonant or electronic excitation.

## 4 Experimental Section

### Nanowire Synthesis

ZnO nanowires with diameters between 200 and 1000 nm and lengths of more than 10 µm were synthesized by the vapor‐liquid‐solid (VLS) growth mechanism in a horizontal tube furnace.^[^ ^33^ ^]^ The cobalt doping was realized by ion implantation with an ion energy of 380 keV.^[^ ^17^ ^]^ Two sets of samples have been prepared with varying implantation conditions: either with an ion fluence of 7.1 × 10^14^ cm^−2^ at 700 °C, or with 1.2 × 10^16^ cm^−2^ at room temperature. The implantation profile was simulated using SRIM (see Figure S1, Supporting Information)^[^ ^34^ ^]^ and results into peak concentrations of 0.05 and 0.7 at%, respectively, at a depth of about 180 nm. Subsequently, the irradiation damage was recovered by annealing the samples in air at 750 °C for 120 min, and single nanowires were imprinted and transferred to clean Si substrates.

### Characterization

Combined nano‐XRF and nano‐XEOL measurements (point and mapping analysis) were performed at the nano‐analysis beamline ID16B of the European Synchrotron Radiation Facility (ESRF) located in Grenoble, France.^[^ ^35^ ^]^ The measurements were conducted at cryogenic temperatures of ≈7 K using a liquid He cooled mini‐cryostat.^[^ ^36^ ^]^ A schematic visualization of the experiment is depicted in Figure 1. The filling mode for the storage ring was the “16 bunch mode,” which corresponds to a period of ≈178 ns between the individual X‐ray pulses, with a duration of about 60 ps FWHM each (see Figure S2, Supporting Information). The energy of the synchrotron beam was set to 17.5 keV in “pink‐beam” mode (Δ*E*/*E* = 10^−2^) with a focal spot size of about 62 × 74 nm^2^ (V × H) measured by knife edge scans (see Figure S3, Supporting Information).^[^ ^35^ ^]^ The photon flux was varied in a range of 3 × 10^10^ to 6 × 10^12^ photons s^−1^ using Si attenuators. The investigated nanowire was raster scanned in a top‐view geometry through the nanofocused hard X‐ray beam with step sizes from 50 to 200 nm and a counting time ranging between 0.5 and 10 s. At each position, a complete spectrum of the emitted XRF radiation was recorded with a three Silicon Drift Diodes (SDD) detector, while the corresponding XEOL signal was simultaneously detected using a spectrometer “Maya” by Ocean Optics/Insights. On a pixel‐by‐pixel basis, the concentration of all elements of interest were individually estimated by fitting the XRF signal using the PyMca software,^[^ ^37^ ^]^ as shown in Figure 1. Due to the absorption of the Kapton window of the cryostat, the air between cryostat and detector and the Be window of the detector, elements lighter than silicon cannot be detected. Additionally, a Hamamatsu StreakScope C14381‐110 camera was set up at the beamline in order to temporally resolve the optical luminescence. The streak camera operated in the photon counting mode that allowed the detection of the low luminescence signals of the cobalt‐doped ZnO nanowires.

## Conflict of Interest

The authors declare no conflict of interest.

## Author Contributions

The manuscript was written through contributions of all authors. All authors have given approval to the final version of the manuscript.

## Supporting information

Supporting Information

## Data Availability

The data that support the findings of this study are available from the corresponding author upon reasonable request.
