# Supplementary material for: Spatially Resolved Dynamics of Cobalt Color Centers in ZnO Nanowires
Source: Adv Sci (Weinh). 2022 Nov 20;10(1):2205304. doi: 10.1002/advs.202205304 (PMC9811436; doi:10.1002/advs.202205304)
Supplement: Supplementary file 1 — Supporting Information [file ADVS-10-2205304-s001.pdf]

ADVANCED SCIENCE
Open Access

Supporting Information

for *Adv. Sci.*, DOI 10.1002/advs.202205304

Spatially Resolved Dynamics of Cobalt Color Centers in ZnO Nanowires

Christian T. Plass, Valentina Bonino, Maurizio Ritzer, Lukas R. Jäger, Vicente Rey-Bakaikoa, Martin Hafermann, Jaime Segura-Ruiz, Gema Martínez-Criado and Carsten Ronning*

Supporting Information to

Spatially Resolved Dynamics of Cobald Color Centers in ZnO Nanowires

Christian T. Plass[1,], Valentina Bonino[2], Maurizio Ritzer[1], Lukas Jäger[1], Martin Hafermann[1], Jaime Segura-Ruiz[2], Gema Martínez-Criado[2,3] and Carsten Ronning[1]*

[1] Institut für Festkörperphysik, Friedrich-Schiller-Universität Jena, Max-Wien-Platz 1, 07743 Jena, Germany

[2] ESRF – The European Synchrotron, 71 Avenue des Martyrs, 38043 Grenoble, France

[3] Instituto de Ciencia de Materiales de Madrid (CSIC), Consejo Superior de Investigaciones Científicas, Calle Sor Juana Inés de la Cruz 3, 28049 Cantoblanco, Madrid, Spain

Keywords: ZnO, nanowires, nano-XRF, nano-XEOL, time-resolved XEOL, color centers

***Corresponding Author:** christian.tobias.plass@uni-jena.de

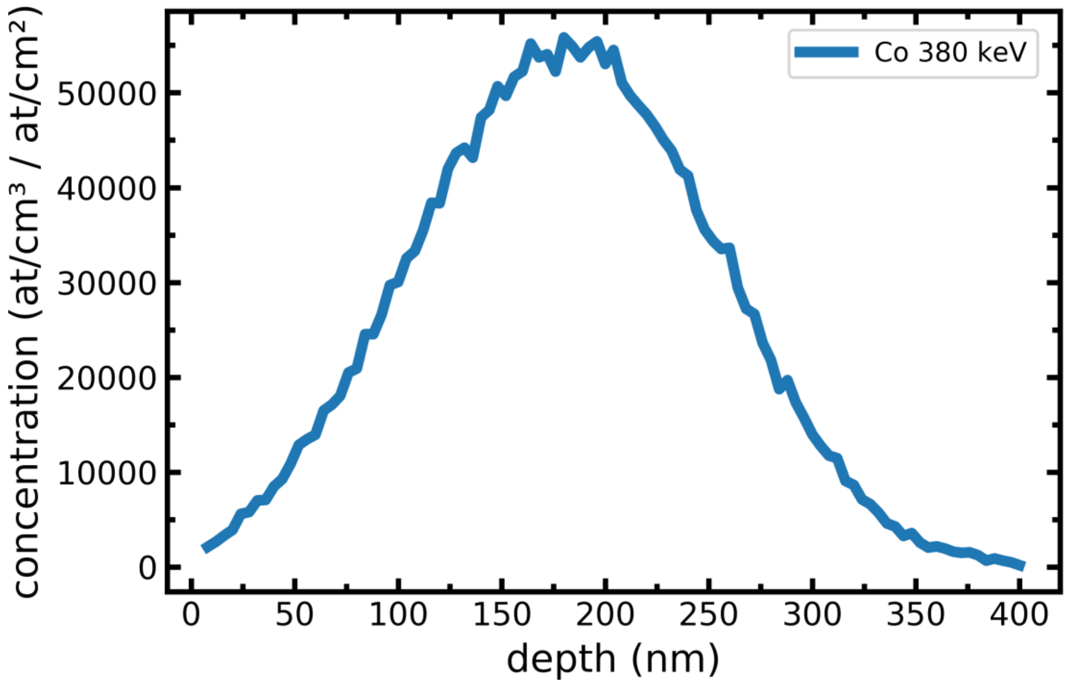

Figure S1. *Implantation profile for Co in ZnO with an energy of 380 keV calculated using SRIM.*

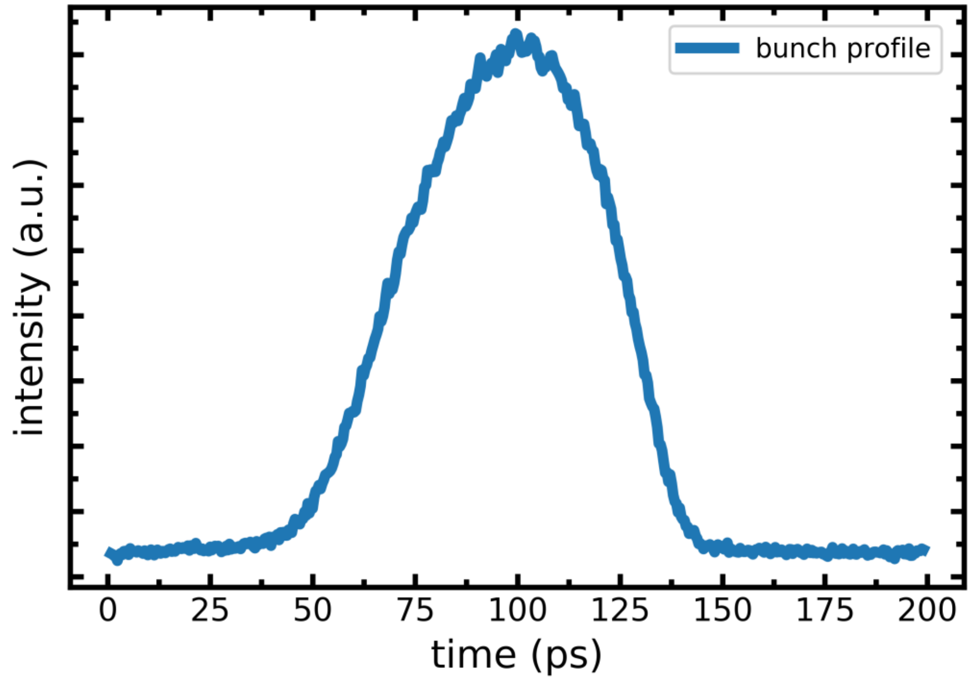

Figure S2. *Beam profile of the X-ray pulses in the "16 bunch mode" at the ESRF.*

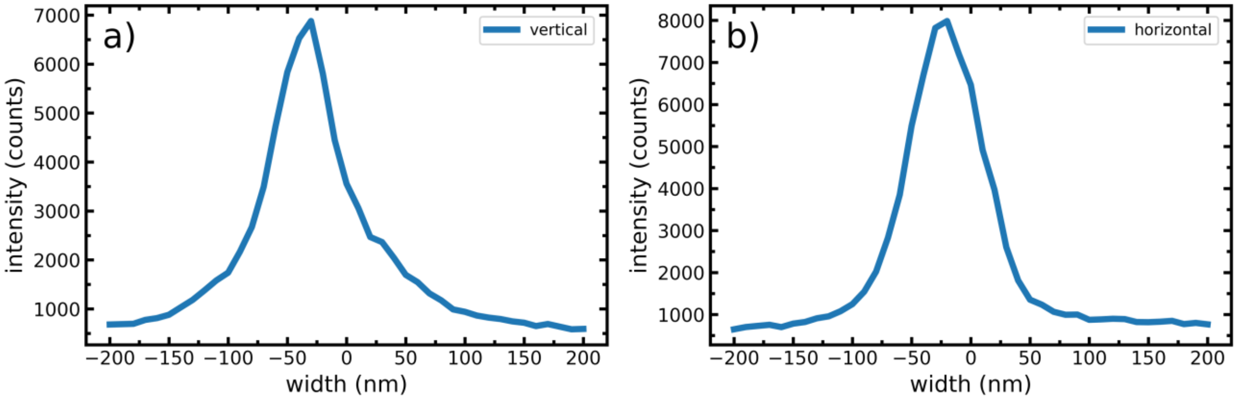

Figure S3. *Profile of the X-ray beam in a) vertical and b) horizontal direction.*

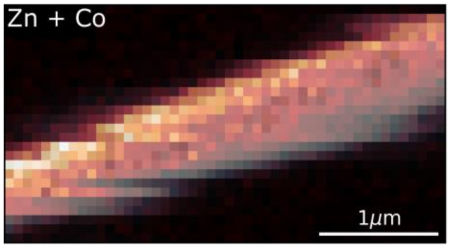

Figure S4. *Overlap of the nanoXRF intensities distributions of Co and Zn (according to figure 2). The color schemes are kept the same for both overlaid images.*

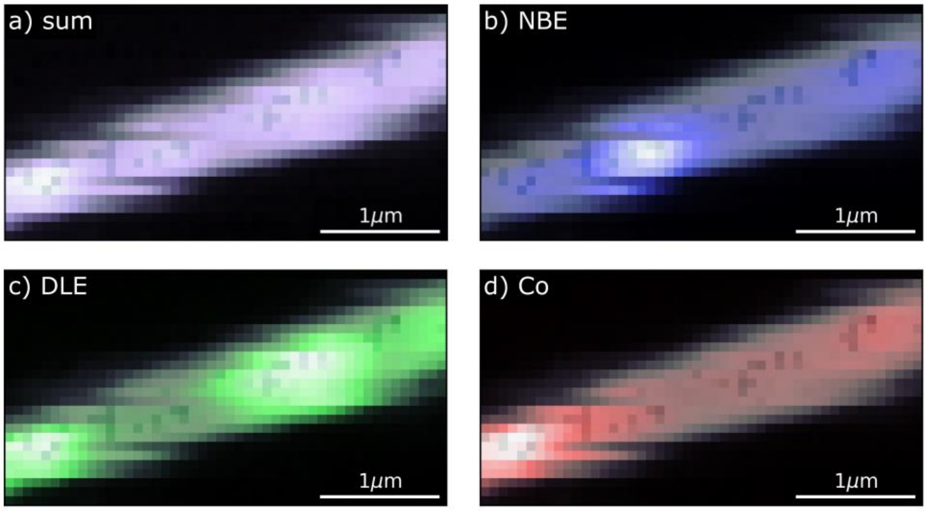

__Figure S5.__ Overlap of the different spectral features of the optical emission (according to figure 3) and the Zn distribution (according to figure 2). The color schemes are always kept the same for both overlaid images.

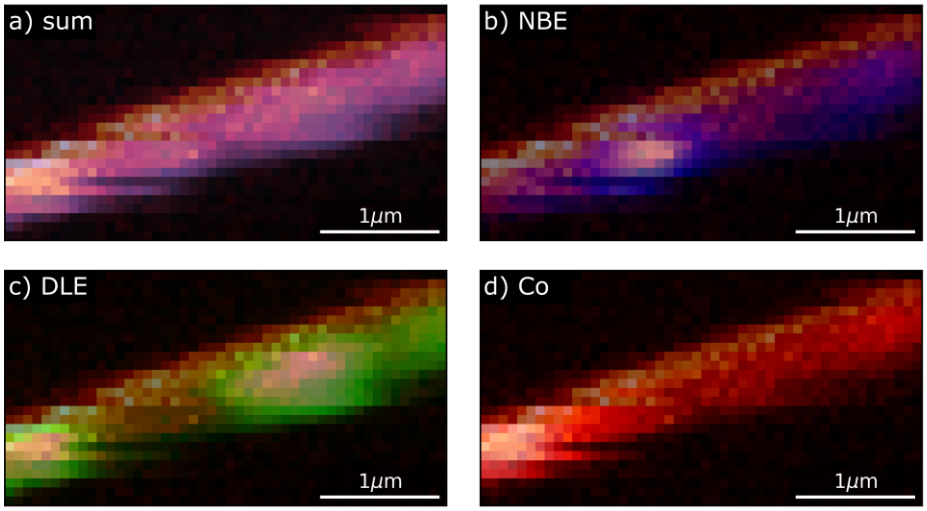

__Figure S6.__ Overlap of the different spectral features of the emission (according to figure 3) and the Co distribution (according to figure 2). The color schemes are always kept the same for both overlaid images.

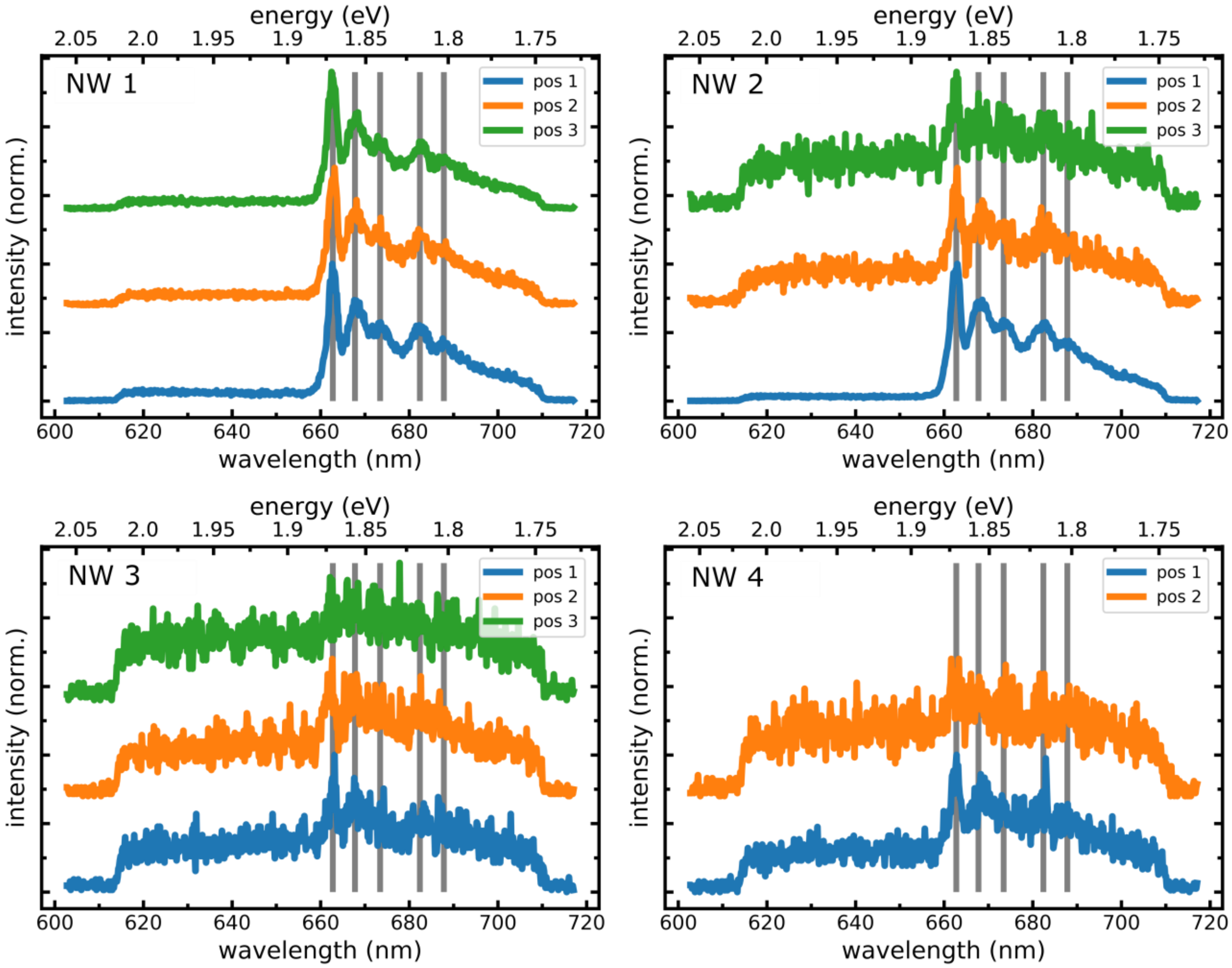

Figure S7. *XEOL yield in the spectral range of the Co emission detected with the streak camera at different positions on several nanowires with a nominal Co concentration of 0.05 at.%.*

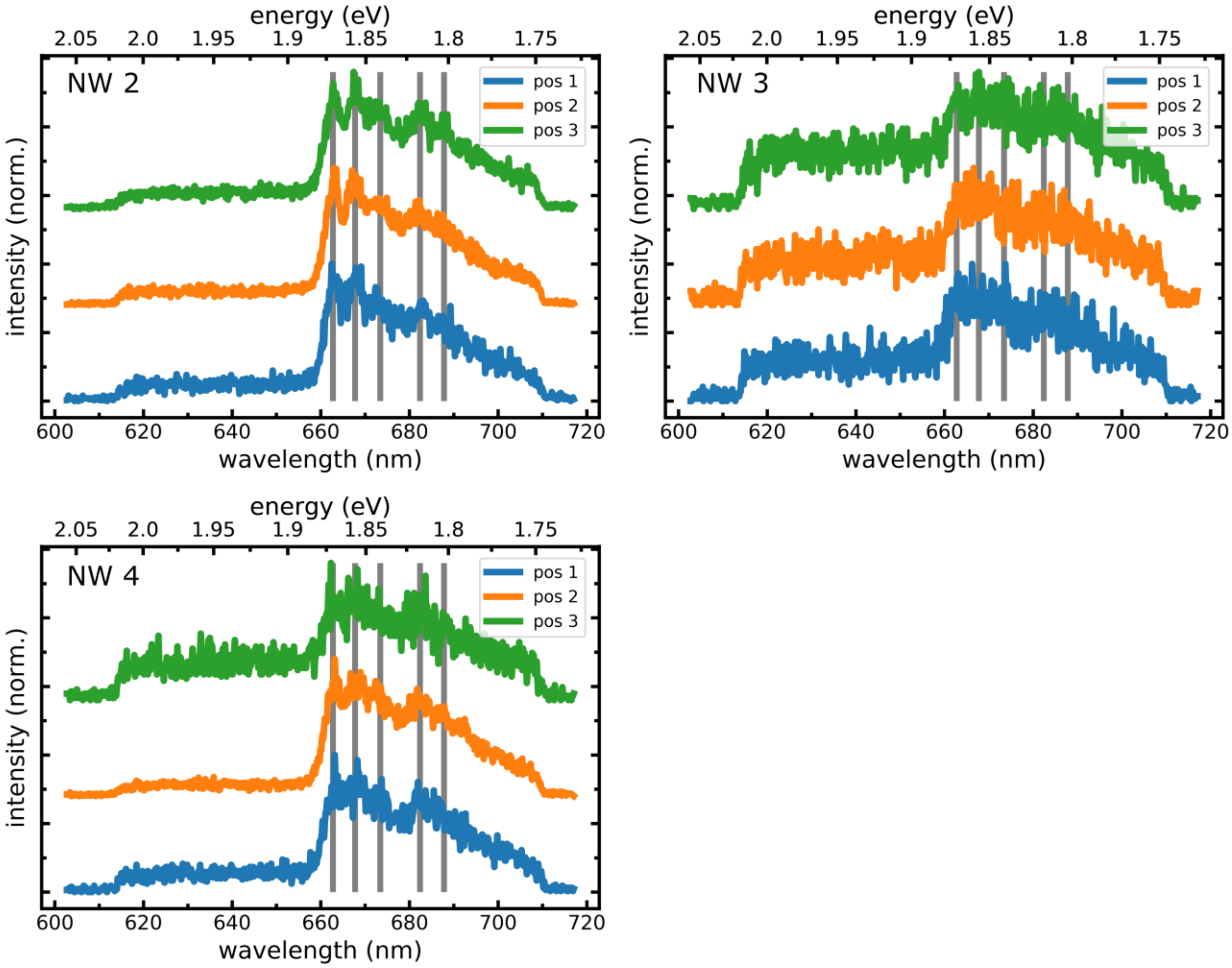

Figure S8. *XEOL yield in the spectral range of the Co emission detected with the streak camera at different positions on several nanowires with a nominal Co concentration of 0.7 at.%.*

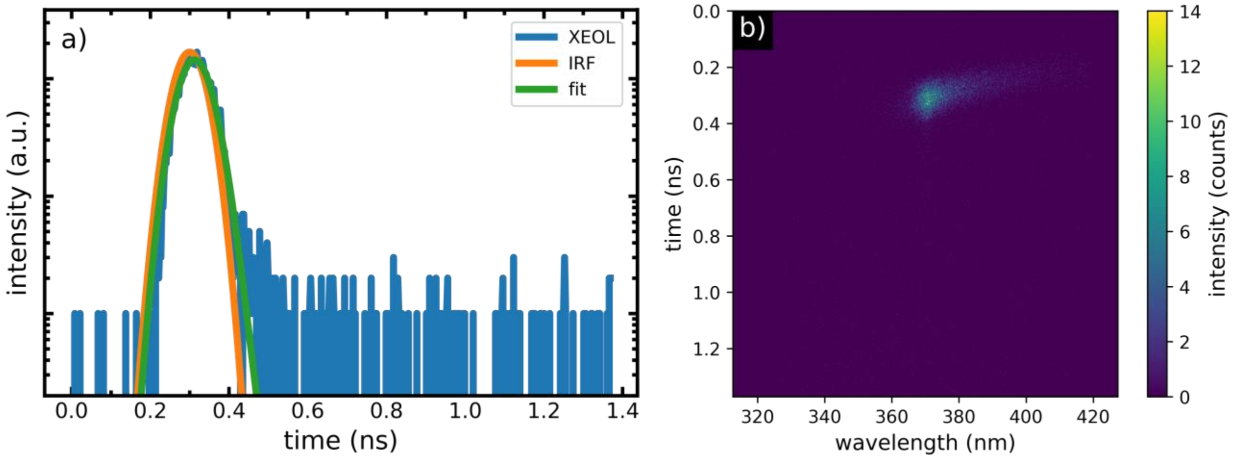

Figure S9. *Time-resolved XEOL measurements of the NBE of a nanowire with a low intensity / short decay. (a) Scan along the time axis of the (b) spectrally resolved streak image according to the area marked in figure 4a.*

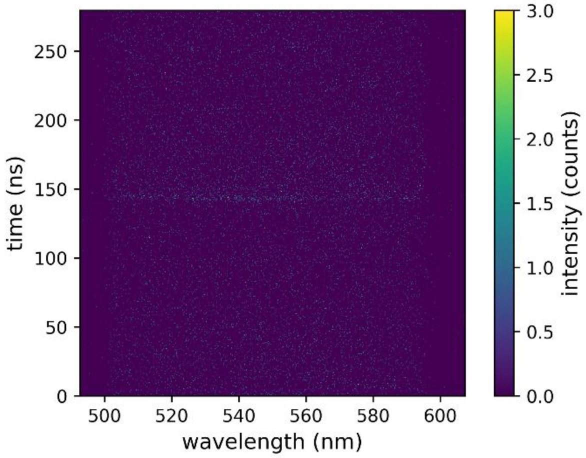

Figure S10. *Time-resolved XEOL measurements of the DLE of the nanowire discussed in this work.*

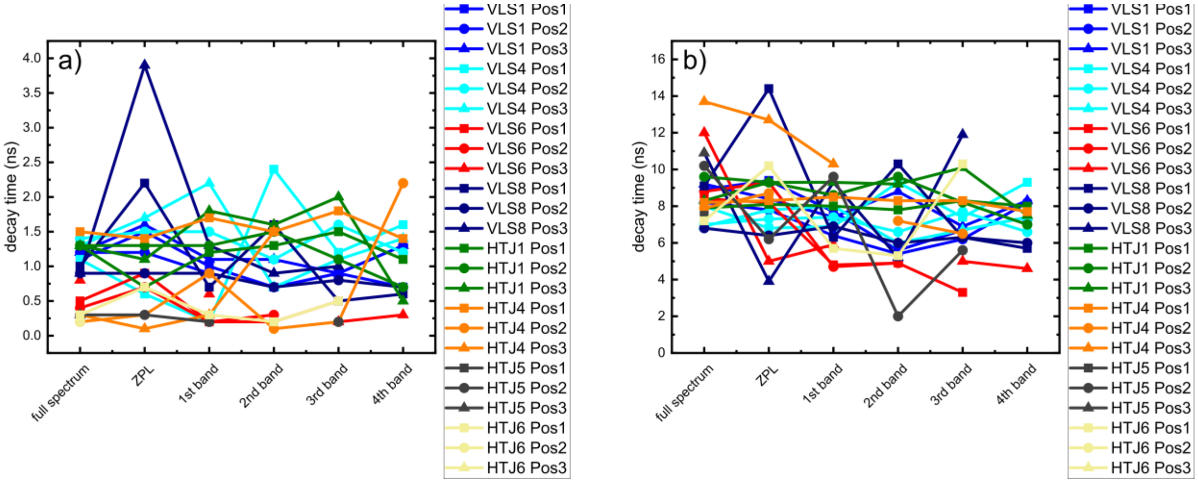

Figure S11. *Decay times of the different phonon lines for different positions on the investigated nanowires for (a) the fast decay and (b) the slower decay.*

Detailed Description of the Optical Path for the XEOL Signal

A schematic drawing of the collection of the XEOL signal can be found in the figure S12. The sample is placed in a mini-cryostat36 with a diamond window (400 µm thickness), which lets the X-ray beam as well as the optical luminescence pass through. The luminescence light is then collimated by a parabolic mirror (Thorlabs MPD019-H23-F01-SP-2). It is then focused on to an optical fiber (Ocean Insight QP50-2-UV-BX) by another parabolic mirror (Thorlabs RC12SMA-P01 or -P01, depending on the wavelength). The optical fiber is on the other end connected to an identical parabolic mirror (Thorlabs RC12SMA-P01) mounted on a 30mm cage system (Thorlabs), to collimate the light. Then it is focused onto the entrance slit of the spectrograph (Andor Kymera 328i) by an achromatic lens (Thorlabs AC254-200-AB-ML). The spectrally dispersed light is finally focused onto the slit of the streak camera (Hamamatsu StreakScope C14381-110), using the focusing mirror of the spectrograph. When measuring time integrated

XEOL, the optical fiber is directly connected to a mini-spectrometer “Maya” by Ocean Optics/Insights.

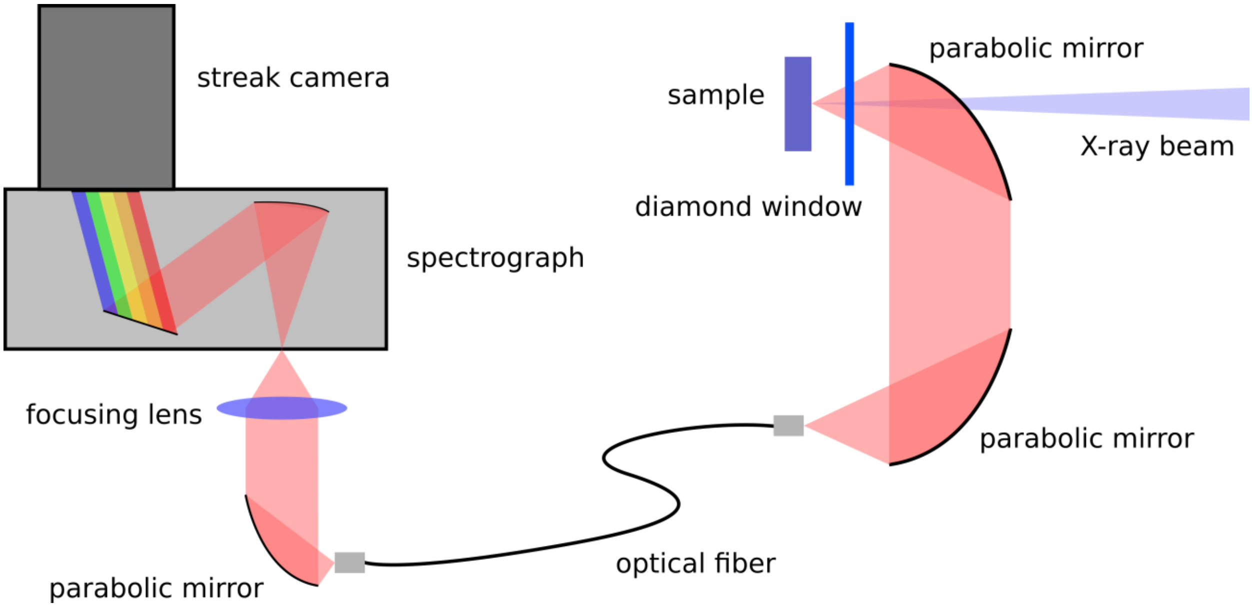

Figure S12. *Schematic drawing of the optical setup for the XEOL collection using the TR-XEOL setup.*
